# Polylactide Films with the Addition of Olive Leaf Extract—Physico-Chemical Characterization

**DOI:** 10.3390/ma14247623

**Published:** 2021-12-11

**Authors:** Sylwia Grabska-Zielińska, Magdalena Gierszewska, Ewa Olewnik-Kruszkowska, Mohamed Bouaziz

**Affiliations:** 1 Department of Physical Chemistry and Physicochemistry of Polymers, Faculty of Chemistry, Nicolaus Copernicus University in Toruń, Gagarin 7 Street, 87-100 Toruń, Poland; olewnik@umk.pl; 2 Electrochemistry and Environmental Laboratory, National Engineering School of Sfax, University of Sfax, BP1173, Sfax 3038, Tunisia; mohamed.bouaziz@fsg.rnu.tn

**Keywords:** polylactide films, olive leaves, extract, food packaging, antioxidants

## Abstract

The aim of this work was to obtain and characterize polylactide films (PLA) with the addition of poly(ethylene glycol) (PEG) as a plasticizer and chloroformic olive leaf extract (OLE). The composition of OLE was characterized by LC-MS/MS techniques. The films with the potential for using in the food packaging industry were prepared using a solvent evaporation method. The total content of the phenolic compounds and DPPH radical scavenging assay of all the obtained materials have been tested. Attenuated Total Reflectance-Fourier Transform Infrared Spectroscopy (FTIR-ATR) allows for determining the molecular structure, while Scanning Electron Microscopy (SEM) indicated differences in the films’ surface morphology. Among other crucial properties, mechanical properties, thickness, degree of crystallinity, water vapor permeation rate (WVPR), and color change have also been evaluated. The results showed that OLE contains numerous active substances, including phenolic compounds, and PLA/PEG/OLE films are characterized by improved antioxidant properties. The OLE addition into PLA/PEG increases the material crystallinity, while the WVPR values remain almost unaffected. From these studies, significant insight was gained into the possibility of the application of chloroform as a solvent for both olive leaf extraction and for the preparation of OLE, PLA, and PEG-containing film-forming solutions. Finally, evaporation of the solvent from OLE can be omitted.

## 1. Introduction

Currently, the world is flooded with packaging that is not environmentally friendly. Therefore, in the process of producing packaging, especially packaging intended for food, more attention is being paid to packaging based on biodegradable polymers. Such materials include polylactide, which is easily decomposed in the presence of microorganisms or water, and at the same time, remains stable during exploitation. In addition to the biodegradability of packaging, materials characterized by antioxidative properties are also sought after, which are capable of extending the shelf life of products.

Active packaging includes systems that absorb or emit substances [1,2]. The absorbers often eliminate unwanted substances such as oxygen, ethylene, excess moisture, and certain smells and flavors. In contrast, the emitters exude substances with an antimicrobial or antioxidative activity. It should be emphasized that active packaging is a system that modifies the composition of food or interacts with it. Recent studies have allowed for the addition of natural extracts that can act as antioxidants, flavor and odor absorbents, and enzymatic and antimicrobial agents [3,4]. Fat oxidation is widely recognized as one of the most important mechanisms leading to the deterioration of foods containing triglycerides. Lipid oxidation leads to a shortening of the shelf life of food due to the highly undesirable, unfavorable changes in taste and/or odor, and to the deterioration in texture and nutritional quality [5]. For this reason, certain natural substances with antioxidative properties are incorporated into the package matrix.

The most popular groups of antimicrobial and antioxidative compounds incorporated into polymeric materials include essential oils, plant-derived substances, and other organic compounds. Essential oils that are incorporated into the polymer matrix because of their antioxidative or bactericidal properties comprise cinnamon, rosemary oil and clove [6,7,8,9], eugenol, [10,11,12], curcumin oil [13], and tea tree oil or tea seed oil [14,15], as well as oregano oil [16] or *Melaleuca alternifolia* essential oil [14]. The introduction of essential oil into the polymer matrix, in most cases, improves elongation at the break of the obtained materials, reduces the water vapor permeation rate, and results in antibacterial or antioxidative properties.

The other popular group of compounds characterized by antioxidative properties are flavonoids, with quercetin, catechin, and its derivatives being the most widely applied [17,18,19,20]. The flavonoids mentioned above are known as valuable antioxidative and antimicrobial substances. Moreover, they can be used as natural stabilizers and color indicators [21].

Other natural alternatives include different plant extracts. In the work of Ji-Hee Kim et al. [22], the antioxidative activity of extracts originating from the coffee residue in raw and cooked meat was studied. Different extracts were obtained using *Limnophila aromatica,* which is commonly used as a spice and a medicinal herb [23]. The results indicated that *L. aromatica* reduces oxidative stress and can be used in dietary applications. In the work of Badakhshan Mahdi-Pour [24], the antioxidative activity of methanol extracts obtained from different parts of *Lantana camara* was studied. The antioxidative effect of coconut shell extract was proven in the work of Tanwar et al. [25], while Martínez et al. [26] investigated the antioxidative and antimicrobial activity of rosemary, pomegranate, and olive extracts in fish patties. Currently, scientists have started to thoroughly study polymeric materials containing plant extracts [27,28,29,30]. Active packaging films consisting of polymer and a plant extract have started to be extremely attractive. This phenomenon is related to the natural origination of extracts, their antibacterial and antioxidative properties, as well as their compatibility with the polymer matrix and the availability of plants—the source of the extracts [31]. The preparation and antioxidative activities of gelatin films incorporated with Ginkgo biloba extract were described in the work of Hu et al. [32]. García et al. [33] analyzed films made of poly(ε-caprolactone) with the addition of almond skin extract.

Olive leaf extract is likely to be a promising antibacterial and antioxidative additive. It is well known that olive leaf extract contains polyphenols, namely oleuropein, hydroxytyrosol, tyrosol, verbacoside, rutin, apigenin-7-glucoside, and luteolin-7-glucoside [34]. In most cases, active compounds present in the olive leaves are extracted with different solvents. The type of used solvent constitutes a crucial factor on which the efficiency of the extraction depends. In the case of olive leaves the effectiveness of the following solvents and its mixtures has been studied: water [30,35], methanol-water (4:1 *v*/*v*) as well as (80:20, *v*/*v*) mixtures [36,37], ethanol [38] chloroform-methanol (50/50 *v*/*v*) mixture [34,39], acetone, ethanol and their aqueous forms (10–90%, *v*/*v*) [40], petroleum ether, dichloromethane, methanol [41]. Taking into account the antibacterial and antioxidative properties of olive leaf extracts, some researchers introduced them into the polymer matrix to obtain active packaging films. The effectiveness of antimicrobial packaging based on the carrageenan filled with olive leaf extract has been studied in the work of Thamiris Renata Martiny [30]. It was established that the olive leaf extracted in water allowed to obtain polymeric films highly effective against *E. coli*. In the work of Erdohan [39], olive leaf extract obtained in the mixture chloroform-methanol (50/50 *v*/*v*) was introduced into the polylactide-based film containing glycerol as the plasticizer. However, it should be stressed that polymeric films were formed from chloroform-ethanol mixtures. Introduction of aqua olive leaf extract into the carrageenan matrix significantly decreases elastic modulus of the obtained materials and simultaneously increases the value of elongation at break. Moreover, the obtained results show that the incorporation of the OLE into the polymeric film leads to an increase in the WVPR [30]. In the work of Erdohan et al. [39], polylactide with an addition of glycerol as plasticizer and different olive leaf extracts was analyzed. Based on the obtained data, it was established that the ethanol concentration on the solvent mixture significantly affected the WVPR as well as the mechanical properties. Incorporation of the olive leaf extract into the gelatin matrix improves antimicrobial and antioxidant activities of the films, however, it also increases the WVPR of the obtained films [42]. The same tendency was observed in the work of Silveira da Rosa et al. [43]. Moreover, a decrease in the Young’s Modulus was also observed. It is well known that polylactide is not soluble in methanol or ethanol. The introduction of PLA and additives into the mixture of chloroform and methanol leads to the formation of an inhomogeneous structure. For this reason, the objective of our study was to develop PLA-based films with an addition of PEG as a plasticizer and olive leaf extract obtained in chloroform. The proposed method allows omitting the one step in the film-forming procedure, i.e., evaporation of solvent from OLE. The present study analyses the structural (FTIR and SEM), mechanical, and antioxidative properties of olive leaf extract incorporated PLA-based packaging materials. Moreover, the water vapor permeation rate and changes in colour were evaluated.

## 2. Materials and Methods

Acetone and chloroform were purchased from STANLAB (Lublin, Poland). Polylactide (type 2002D, average molecular weight = 79 kDa) was delivered in the form of pellets by Nature Works^®^ (Minnetonka, MN, USA). Poly(ethylene glycol) (M_w_ = 1500 g·mol^−1^) was purchased from Sigma-Aldrich (Steinheim, Germany).

### 2.1. Phenolic Analysis of Olive Leaf Extract

LC-MS/MS analysis was used to examine the olive leaf extract composition.

Olive leaves of the *O. europaea* tree (cultivar Chemlali) were collected from Sfax (Tunisia) in July 2019 and dried for 3 min in a JN-100 microwave dryer (Adasen, Jinan, China) (1200 W). The dry leaves were milled and stored at 4 °C. The extract was prepared from 2.5 wt.% olive leaf powder dispersion in chloroform, and was stirred for 1 h at 30 °C. The obtained solution was filtered using filter paper (Filtres RS, 8–11 mm, Madrid, Spain), and the residual solvent was evaporated at 40 °C under a vacuum. The obtained extract was freeze-dried and stored at 18 °C for the phenolic analysis.

The LC-UV-MS/MS analyses of the obtained extract were performed according to the method described in detail elsewhere [44].

### 2.2. Fabrication of Films

Thin films were obtained using the solvent evaporation method. First, the leaves from the olive tree (Tunisia) were harvested and dried. Then, they were ground in an electric pulverizer (IKA Werke GmbH and Co. KG, Staufen, Germany). An appropriate amount of olive leaf dust was poured with 50 cm^3^ of chloroform, and the resulting mixture was mixed with a magnetic stirrer (Wigo, Pruszków, Poland) for 3 h and filtered. Then, 1, 3, and 5 wt.% extracts were prepared. Then, 1.5 g of polylactide was added to the olive leaf chloroformic extract and mixed 3 h with a magnetic stirrer. Then, 5 wt.% of poly(ethylene glycol), relative to polylactide, was added to the obtained solution and mixed with a magnetic stirrer to reach a homogenous mixture. Poly(ethylene glycol) was used as a plasticizer. The final solution was poured onto a glass petri dish with a known constant diameter and was evaporated at room temperature. The obtained films samples were dried 24 h at room temperature. Polylactide with a poly(ethylene glycol) film was used as a control sample.

### 2.3. The Total Content of Phenolic Compounds

The total content of phenolic compounds was evaluated using the Folin–Ciocâlteu method, with gallic acid as the standard, according to the Malik and Bradford procedure, with slight modifications [45]. The Folin–Ciocâlteu reagent was used as an oxidizing agent. The samples were immersed in a mixture of 0.5 mL of Folin−Ciocalteu reagent, 1 mL of Na_2_CO_3_, and 8.5 mL of distilled water. The samples were incubated at 40 °C in the dark for 30 min. Next, the absorbance of the samples was measured spectrophotometrically at 725 nm using a UV-1800 spectrophotometer (UV-1800, Shimadzu, Reinach, Switzerland). The experiment was run in triplicate, and the results were expressed as a gallic acid equivalent using a five-point calibration curve as mg/mL.

### 2.4. DPPH Radical Scavenging Assay

The free radical scavenging activity of the PLA-based samples was measured in vitro using a DPPH reagent (2,2′-diphenyl-1-picrylhydrazyl, free radical, 95%; Alfa Aesar, Karlsruhe, Germany) [46]. First, 250 µM methanolic solution of DPPH was prepared. Next, small pieces (1 × 1 cm^2^) of films were prepared and placed in a 12-well plate. Then, 2 mL of a DPPH solution was added and the samples were kept for 60 min in the dark. As a control sample a DPPH solution left on the plate without contact with the films was used. After incubation, the absorbance was measured spectrophotometrically at 517 nm (UV-1800, Shimadzu, Reinach, Switzerland). All of the experiments were replicated five times. The antioxidant activity (*RSA*) was calculated using the following formula:

(1) $$RSA=\frac{A_{0}-A_{PB}}{A_{0}}\cdot 100\%,$$

where *A*_0_ is the average absorbance of the DPPH solution without contact with the films, and *A_PB_* is the average absorbance of the DPPH solution after contact with the film being tested.

### 2.5. FTIR-ATR Spectroscopy

Fourier transform infrared spectroscopy with attenuated total reflectance (FTIR-ATR) was used to evaluate the chemical structure of the obtained films. A Nicolet iS10 spectrophotometer (Nicolet iS10, ThermoFisherScientific, Waltham, MA, USA) with a germanium crystal was used for spectra recording. All of the spectra were recorded with the 64 scans and a resolution of 4 cm^−1^, and were evaluated in the range of 400–4000 cm^−1^.

### 2.6. Differential Scanning Calorimetry

Thermal analyses of all PLA-based films were performed using DSC equipment (DSC 204 F1 Phoenix, Netzsch, Germany) in the nitrogen atmosphere (gas flow 20 mL/min). Samples of ca. 3–5 mg were sealed in standard aluminum pans, then heated with a heating rate of 10 °C/min from 20 to 180 °C, cooled to 30 °C, equilibrated for 5 min at 30°, and heated again to 180 °C. The recorded data were analyzed with NETZSCH Proteus software (Version 6.1.0), and the degree of crystallinity ($X_{C}$) was calculated with the following equation [47]:

(2) $$X_{C}=\frac{\Delta H_{F}}{\Delta H^{o}\cdot X_{PLA}}\cdot 100,$$

where $\Delta H_{F}$ is the heat of fusion of the analyzed sample representing the difference between PLA melting enthalpy ($\Delta H_{m}$) and enthalpy of cold crystallization ($\Delta H_{cc}$), $\Delta H^{o}$ is the heat of fusion of a fully (100%) crystalline PLA ($\Delta H^{o}$ = 109 J/g [48]), and $X_{PLA}$ is the mass fraction of the PLA in the film.

### 2.7. Mechanical Properties and Thickness

The mechanical properties were analyzed at room temperature in the dry state using Zwick&Roell 0.5 testing machine (Zwick&Roell Group, Ulm, Germany). Mechanical properties were studied at a crosshead speed of 5 mm/min. The samples were cut using the same bone-shaper (50 mm length, 10 mm width).

The thickness of the samples was measured with an ultrameter type A-91 (Manufacture of Electronic Devices, Warsaw, Poland). For each kind of film, at least five samples were tested.

### 2.8. Water Vapor Permeation Rate (WVPR)

The water vapor permeation rate (WVPR) of PLA-based films was investigated. Three independent tests were performed for each film. First of all, the fresh desiccant was prepared (CaCl_2_ dried at 110 °C for 24 h). Plastic containers of 24 mm in diameter containing a determined amount of desiccant were prepared, with the top covered tightly with the tested films. The containers with CaCl_2_ but without covers were used as the control samples. The containers were placed in an oven at 30 °C with 75% relative humidity. After each 24 h to 7 days, the moisture penetration was determined based on the changes in the desiccant weight. The slopes of the steady-state (linear) portion of weight loss versus time curves were used to calculate the WVPR, according to the following equation:

(3) $$\mathrm{WVPR}=\frac{W}{A\cdot t}\left[g\cdot m^{-2}\cdot h^{-1}\right],$$

where *W* is the weight gained [g], *A* is the area of the film cover [m^2^], and *t* is time [h].

### 2.9. Colour Change

The influence of OLE incorporation and the swelling process in the media of different pH on the color of the obtained films was studied using the MICRO-COLOR II LCM 6 (Dr. Lange, Berlin, Germany) colorimeter. The as-obtained films were immersed in 0.1 M HCL, 0.1 M NaOH, and distilled water for 7 days [46]. Then, films were taken out from the liquid and allowed to dry at room conditions (temperature and humidity). The color parameters of the dry as-prepared (before immersion) and the post-swelled (after immersion) films were measured. The CIE L*a*b* system was applied to calculate the color difference (Δ*E*) of the materials. To establish the total values of color difference (Δ*E*) and color intensity (*C*), the following formulas were used:

(4) $$\Delta E=\sqrt{{\left(L-L^{*}\right)}^{2}+{\left(a-a^{*}\right)}^{2}+{\left(b-b^{*}\right)}^{2}},$$

(5) $$C=\sqrt{{\left(a\right)}^{2}+{\left(b\right)}^{2}},$$

where:

*L*—the component describing lightness,
*a*—represents the color ranging from green (−a) to red (+a),
*b*—represents the color ranging from blue (−b) to yellow (+b),
L*, a*, b*—values characteristic for the control film (PLA/PEG), L* (lightness), a* (redness/greenness), and b* (yellowness/blueness).

Yellowness index (*YI*) was calculated using the following equation [49]:

(6) $$YI=\frac{142.86\cdot b}{L},$$

Each sample was analyzed five times and the mean values were calculated.

### 2.10. Morphology Observations—Scanning Electron Microscopy

Scanning Electron Microscopy was used to observe the surface and cross-section of the obtained films. Before each analysis, the surfaces of the studied films were sputtered with an Au thin layer. Photographs were taken using the Quanta 3D FEG scanning electron microscope (SEM, FEI Company, Hillsboro, OR, USA).

### 2.11. Statistical Analysis

The obtained data were statistically analysed using commercial software (GraphPad Prism 8.0.1.244, GraphPad Software, San Diego, CA, USA). The results, presented as a mean ± standard deviation (SD), were compared using one-way analysis of variance (one-way ANOVA). Multiple comparisons between the means were performed with the statistical significance set at *p* ≤ 0.05. The results from the total content of phenolic compounds, the free radical scavenging activity, the mechanical properties, and thickness and water vapor permeation rate tests were subjected to statistical analysis.

## 3. Results and Discussion

### 3.1. The Total Content of Phenolic Compounds and DPPH Radical Scavenging Assay

There are several studies devoted to the extraction of olive waste and to the determination of the phenolic compound composition for the obtained extracts [40,50,51]. The analysis of these results indicate clearly that the presence of different phenolic compounds in olive leaf extract (OLE) depends on several factors [40,50,51,52]: olive tree cultivation, season, time of extraction, and solvent used. Thus, it is crucial to evaluate the composition of the particularly obtained OLE. Based on the LC-MS/MS analysis, the olive leaf extract composition was determined and is listed in Table 1.

As can be seen, several phenolic compounds were detected in the extract. Oleuropein is the major phenolic compound in olive leaves. Its content varies from 17% to 23% depending upon the harvesting time of the leaves [53]. Among others substances present in chloroformic olive leaf extract, gallic acid, hydroxytyrosol, caffeic acid, rutin, verbascoside, luteolin 7-*O*-glucoside, oleuropein hexoside I, isoverbascoside, apigenin 7-*O*-glucoside, 6′-*O*-[(2E)-2,6-dimethyl-8-hydroxy-2-octenoyloxy]-secologanoside, and jaspolyoside III, were also found. All of the compounds given in Table 1 were previously reported in olive leaf extracts [54,55].

The total content of the phenolic compounds and the free radical scavenging activity of the obtained films with the olive leaf active substances extracted are shown in Table 2. It is well known that phenolic compounds can be found in all parts of the olive plant [56]. It is frequently reported that oleuropein is the most prominent and predominant phenolic compound in olive cultivars and can reach a concentration of 60–90 mg/g in dry leaves [57,58]. The quantitative analysis revealed the presence of phenolic compounds in each type of film, excluding the control film without olive leaf extract. As the concentration of olive leaf extract increases, the phenolic compound content increases, but no significant statistical difference (*p* ≤ 0.05) was observed between PLA/PEG/1OLE and PLA/PEG/3OLE. The film with the highest content of OLE (PLA/PEG/5OLE) was also characterized by the highest phenolic compound concentration (0.637 ± 0.061 mg/mL). This trend is in line with expectations and with the results of other scientists. Da Rosa et al. [44] prepared biodegradable carrageenan films containing olive leaf extract by the evaporation method. They noticed a higher amount of phenolic compounds with the increasing amount of olive leaf extract. Albertos et al. [43] studied olive leaf-gelatin film properties and evaluated that the total soluble phenolic compounds content was higher for materials with a higher extract content.

Taking into consideration the free-radical scavenging (*RSA*) activity, it can be seen that polylactide film with the addition of olive leaf extract was characterized by a higher *RSA* than the non-modified polylactide film. These results prove that the obtained films with the addition of olive leaf extract exhibit improved antioxidant properties. However, it can also be observed that there were no statistically significant differences between the films with different contents of OLE (Table 2). Many scientists reported that as the active substance or extract concentration increases, the antioxidant activity also increases. Tymczewska et al. [59] reported the same trend—a non-modified film based on gelatin was characterized by lower antioxidant activity than the films modified by various contents of rapeseed meal extracts. Roy and Rhim [60] also noticed that the antioxidant activity increased with the increased concentration of the modifier (in this case, curcumin) in the material. A similar relationship was shown by Dou et al. [61] where gelatin/sodium alginate materials with the addition of various contents of tea tree polyphenols were tested. It can be assumed that free radical scavenging can occur (a) on the film surface and (b) in the solution. The (b) option is strongly dependent on the material swelling and the possible release of active substances. As PLA is not soluble in methanol, PLA-film swelling in this medium is very low and reduces the possibility of OLE components migration into the external solution during *RSA* testing. Due to this, it can be stated that free radical scavenging of PLA/PEG/OLE films is primarily an effect of the surface composition. It should be stressed out that there are minor differences in the total OLE content between PLA/PEG/OLE films prepared in this study. Moreover, as the solvent evaporation method was used, there can be slight differences in the composition of the film surface and the inner parts. All of the reasons mentioned above could be responsible for the minor differences in *RSA* observed for PLA/PEG/OLE films.

### 3.2. FTIR-ATR Spectroscopy

**Figure 1:**
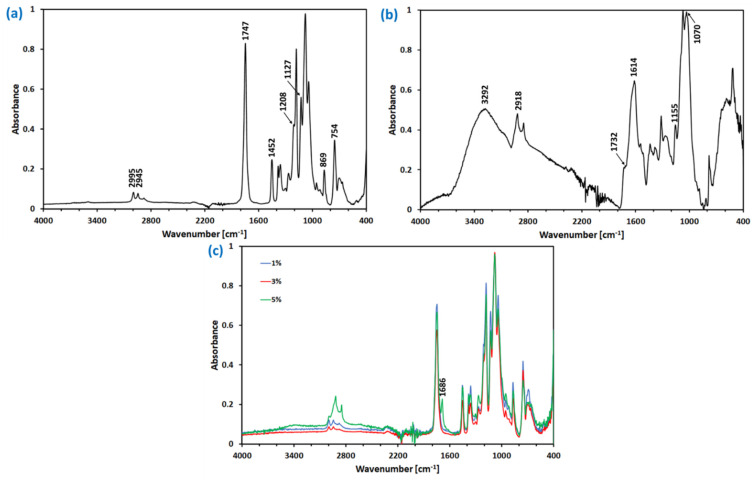
The FTIR-ATR spectra of the control samples (PLA/PEG (**a**) and OLE (**b**)) and polylactide films with the addition of olive leaf extract (**c**).

To evaluate the molecular structure of PLA/PEG and PLA/PEG/OLE films and to confirm the possible interactions between PLA, PEG, and OLE, FTIR-ATR analysis was performed (Figure 1). The molecular structure of the pristine PLA film was already analyzed with the FTIR technique and the results described elsewhere [62], confirming the presence of most characteristic functionals by vibrational bands at: 2999 cm^−1^ and 2945 cm^−1^ (ν_as_ and ν_s_ C-H in -CH_3_), 1763 cm^−1^ (ν_s_ -C=O), 1451 cm^−1^ (bending asymmetrical C-H in _-_CH_3_) 1207 and 1127 cm^−1^ (ν_s_ -C-O-C-), 873 cm^−1^ (ν -C-COO), and 756 cm^−1^ (deformation vibration CO). In the FTIR spectra of PLA/PEG (Figure 1a), almost the same absorption bands as in neat PLA were noticed. The resemblance of PLA and PLA/PEG spectra results from the structural similarity of PLA and PEG. However, as already proven by Yuniarto and coworkers [63], there are slight differences between intensity and bands position between PLA and PLA-PEG films. These confirm that the poly(ethylene glycol) (PEG) hydrophilic group interacts with the polar groups (e.g., C=O) of PLA, resulting in hydrogen bonding.

The spectrum of OLE (Figure 1b) revealed several bands characteristic for the presence of different active compounds listed in Table 1. Most of the OLE components possess characteristic functionals in their structure. Their occurrence was confirmed through the vibrational bands at 3292 and 1732 cm^−1^ (stretching nodes: N-H in amine, O-H in phenols, C=O in carboxylic acid and C=C in alkenes), 1155 cm^−1^ (stretching vibration: C-OH in the protein or C-N in the amine), 2918 cm^−1^ (C-H stretching), 1614 cm^−1^ (indicate the fingerprint region of CO, C-O and O-H, C-O in amide I), and 1070 cm^−1^ (C-N stretching in amine). With a view of the differences in the composition of the OLE extracts connected to the olive leaf sources and processing, the recorded OLE spectra (Figure 1b) remained in agreement with others presented in the literature [64,65,66].

The comparison of the PLA/PEG, OLE, and PLA/PEG/5OLE spectra indicates the characteristic bands of both PLA/PEG and OLE in the final OLE extract-based polymeric films (Figure 1c); however, the most intense bands correspond to PLA/PEG. This can be due to the relatively low amount of OLE in the final film. In particular, the preparation of PLA/PEG films with the usage of OLE extract resulted in a new adsorption band at 1686 cm^−1^. It can be assigned to the ν O-H of the OLE components; however, its position is visibly shifted in comparison to the spectra of pure OLE (i.e., 1614 cm^−1^). Similarly, in the 2800–3100 cm^−1^ region, three bands were noticed: at 2994 cm^−1^ (corresponding to the asymmetric stretching of C-H in -CH_3_ in PLA), 2918, and 2849 cm^−1^ (characteristic for OLE components). These observations allow for the suggestion of possible interactions between the chemicals present in the OLE and PLA/PEG matrix.

### 3.3. Mechanical Properties and Thickness

**Figure 2:**
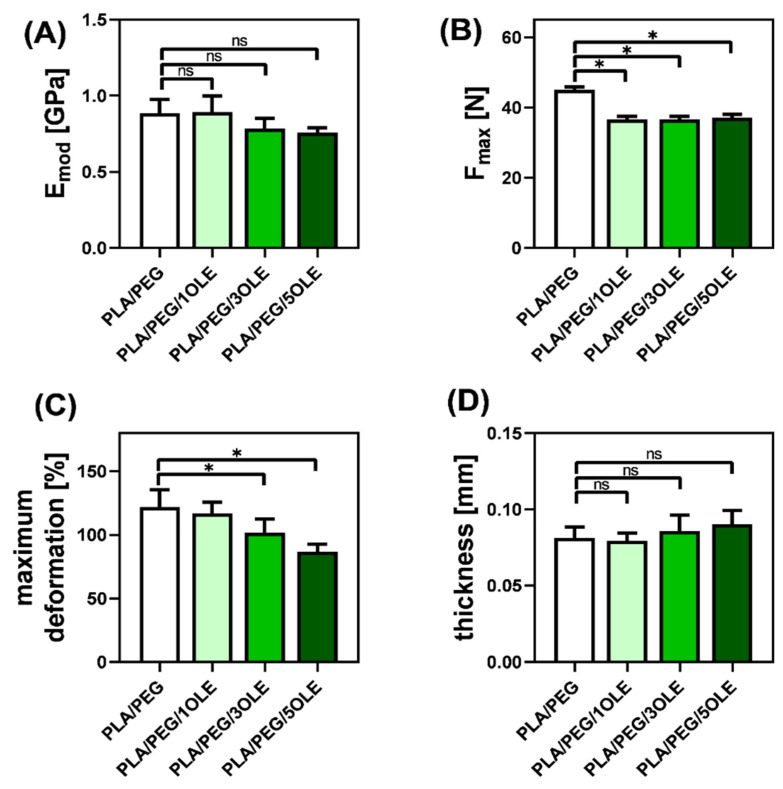
The mechanical properties: (**A**) Young modulus, (**B**) maximum force at break, (**C**) maximum deformation, and (**D**) thickness of the obtained films; *—statistically significant difference vs. control sample (PLA/PEG); ns—no statistically significant difference vs. control sample (PLA/PEG).

A tensile test coupled with thickness measurements was done to estimate the effect of OLE addition on the mechanical properties of PLA/PEG polymeric films. Stress–strain curves were used to evaluate Young’s modulus (E_mod_), elongation at break (Maximum deformation), and maximum force at break (F_max_) (Figure 2).

It was proven several times in the literature [67,68,69], and also by us, that the addition of a PEG plasticizer into the PLA matrix causes an increase in the free volume inside the polymeric film, which in turn facilitates the movement of the polymeric chains. As already indicated in the literature [70], PEG affects the characteristics of PLA by disturbing the intermolecular forces. Thus, neat PLA/PEG films exhibit relatively high elongation at break and a maximum force at break (F_max_) compared to pure PLA. As shown in Figure 2, an introduction of OL extract into the PLA/PEG matrix causes a reduction of maximum deformation, Young’s modulus (E_mod_), and F_max_ values. Moreover, the maximum deformation and E_mod_ depend on the amount of OLE, while F_max_ is almost independent of the OLE concentration. In this context, it can be stated that the incorporation of OLE slightly worsened the mechanical properties of PLA/PEG films.

In general, the literature indicates that the addition of natural essential oils causes an increase in the mechanical properties of pure PLA-based films [71]. Different observations have been made for PEG plasticized PLA materials. Tarach et al. [69] noticed an increase in maximum deformation after incorporating tea tree essential oil into PEG/PLA-based films. Similarly, Chieng et al. [72] registered maximum deformation values for poly(lactic acid) plasticized with poly(ethylene glycol) and epoxidized palm oil in comparison with neat poly(lactic acid) films. In turn, Vasile and coworkers [73] showed a decrease in E_mod_ after the addition of a low amount of rosemary extract into the PLA/PEG (80/20 *w*/*w*), while F_max_ was almost unaffected. This suggests that when OLE is added into an already plasticized PLA-matrix, both components (OLE and plasticizer) can act antagonistically on the polymer chain movement ability, and stay in agreement with the almost invisible changes in the FTIR spectra of PLA/PEG after OLE addition.

It is known that the mechanical properties of PLA-based materials are affected by different polymer properties, as well as by crystallinity [74,75]. In general, it is assumed that the greater the crystallinity, the harder the polymer, as crystallinity reduces the degree of freedom for the molecular chains to move. As we discussed earlier [69], the presence of chloroform within the PLA-based materials provides suitable conditions for the crystallization of the polymer. Moreover, based on the results presented in Table 3, it can be seen that the addition of OLE increases crystallinity, thus causing a reduction of maximum deformation of PLA/PEG/OLE materials. Based on the differences in crystallinity, which slightly changed after OLE incorporation, it can be assumed that OLE components act as a nucleus of crystallization, and finally reduce the maximum deformation. The current findings remain in agreement with the data reported by Malgorzata Latos-Brozio and Anna Masek [76], who proved that plant polyphenols can act as nucleating substances, increasing the crystallinity of PLA, and with the results of Javidi et al. [77], who stated that the essential oil facilitates the process of PLA crystallization.

### 3.4. Water Vapor Permeation Rate (WVPR)

WVTR, according to the definition, is the steady rate at which H_2_O vapor permeates through a barrier at a specific temperature and relative humidity (RH) in a given time. It is a crucial parameter in evaluating the food-package potential, as water affects the product’s quality and shelf-life. Depending on the type of packaged food, WVPR values are expected to be relatively low or high, so there is no “good” value or range for WVPR.

Water barrier properties of PLA/PEG and OLE-containing PLA/PEG films were characterized by calculating the water vapor permeation rate (WVPR) values (Table 3). As can be seen, the application of PEG-plasticized polylactide film substantially reduces the water transport from the environment (of relative humidity RH = 75%) into the applied desiccant. However, the further addition of different amounts of OLE does not significantly affect the WVPR values.

As we already discussed [78], several factors affect the transport properties of polymeric films. The WVPR value depends on both the environmental factors (e.g., temperature, relative humidity, the difference in pressure, or concentration gradient across the film) and the material molecular and supramolecular characteristics (e.g., thickness and area, nature and M_w_ of the polymer, type, and content of additives).

The literature [79,80,81] indicates that, in general, neat PLA films are medium-to-low barrier materials to water vapor. The barrier properties of PLA-based films are highly influenced by their polymer chain orientation and packing, defined through crystallinity, crystal thickness, and morphology [82,83]. As indicated by Drieskens et al. [83], the crystallization of PLA has a positive effect on the barrier properties towards water and oxygen. The decrease of permeability in the case of a more crystalline polymer structure was also already described well by the Maxwell equation [84]. Generally, the reduction of gas and water vapor transport by crystallization can be explained as [83] (a) decrease in the amorphous phase that, opposite to the crystalline phase, is permeable to gases, and (b) increase in the tortuosity. In this context, according to the evaluated degree of crystallinity (Table 3), which increases with the addition and the content of OLE, a reduction of WVPR should be observed.

The reason for such unexpected changes in WVPR values, which do not vary with $X_{C}$, is probably connected with the nature of the additive (olive leaf extract). The primary phenolic compound found in this extract is oleuropein. For its extraction, simultaneously with other phenolic compounds, organic solvents of a higher polarity (e.g., methanol and ethanol) and water are proposed. It can be assumed that those components exhibit a higher affinity toward more polar solvents. Thus, the addition of OLE into the PLA/PEG matrix can cause an increase in water vapor diffusion. To summarize, it can be stated that an increase in the degree of crystallinity and the addition of OLE act antagonistically to the WVPR value. Finally, no substantial differences in WVPR were observed in the case of PLA/PEG and PLA/PEG/OLE films.

A similar effect of OLE addition on water transport properties was observed by Martiny et al. [30] for carrageenan/glycerol films. It should be added that researchers used a significant amount (62.5 wt.%) of OLE based on carrageenan mass. Contrary, García et al. [85] presented a reduction in water vapor permeation with the increasing amount of olive extract (OLE) in corn starch/glycerol films.

It is worth noting that in the above-discussed context, the OLE addition neither improved nor worsened the barrier properties of the PLA material toward the water.

### 3.5. Colour Change

**Figure 3:**
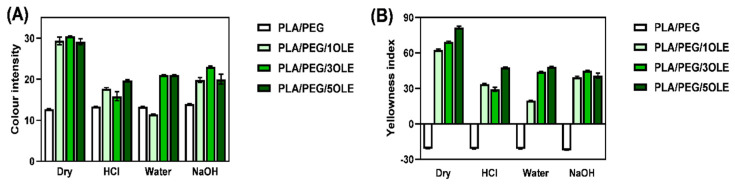
Color intensity (**A**) and Yellowness index (**B**) of PLA/PEG and PLA/PEG/OLE films.

In Table 4, CIEL*a*b* *L*, *a*, and *b* color parameters, as well as total color difference (Δ*E*) of PEG-PLA and OLE-doped PEG-PLA as-prepared (dry) and post-swelled films, are given. Moreover, the calculated color intensity and yellowness index (*YI*) are presented in Figure 3.

All of the films were found to be visually homogeneous, slightly opaque, with a shade of blue or green. As shown (Table 4), the dry PLA/PEG film is characterized by *a* value close to 0 and *b* = −12.86 with a high *L* = 86.46 ± 0.05 parameter. These data correlate with those presented by us earlier for PLA films plasticized with PEG [18], and indicate the white/light blue color of this film. The usage of olive leaf extracts in PLA film preparation resulted in polymeric materials (PLA/PEG/OLE) of almost an unaffected *a* value, but substantially contrasting *L* and *b* values. The higher the concentration of OLE, the lower *L* value that was measured, indicating reduced lightness of films, changing from 66.64 ± 1.14 to 50.28 ± 0.60, for PLA/PEG/1OLE to PLA/PEG/5OLE, respectively. The usage of OLE causes the *b* color parameter to change the sign from slightly negative (for PLA/PEG) to positive (*b* ≈ 30 for PLA/PEG/OLE). All changes in the *a*, *b,* and *L* parameters confirm the visible color change (Δ*E* > 5—observer notices two different colors) from light blue to dark green (“olive”) (visualization in Table 4). The obtained results are consistent with the color of the olive leaf extracts (see Figure S3 in Supplementary Materials).

The human eye functions as a spectrometer analyzing the light reflected from the surface of a solid or passing through a liquid. If we consider white light as composed of a broad range of radiation wavelengths in the ultraviolet (UV), visible, and infrared (IR) portions of the spectrum, characterized by a wavelength, green color is observed when the substance absorbs in the red (780–620 nm) and the blue (495–455 nm), and green-blue when the substance absorbs orange-red (595–750 nm) [86,87].

Most of the colour organic substances belonging to pigments contain certain groups called chromophores [88]. Olive leaves’ colour depends on the pigment composition and concentration, especially with reference to chlorophyll. As it was already given by Imen Tarchoune [89], the green colour of olive leaves and its extracts results from the presence of chlorophylls (absorbance 646–664 nm) and carotenoids (absorbance at 480 nm). As shown in Figure S3 (Supplementary Materials), the higher amount of olive leaf dust used, the more intense the green colour of extract observed. However, even if the colour intensity of PLA/PEG/OLE films was found higher in reflection to PLA/PEG, there is no visible correlation between the colour of extracts and color intensity of resulting PLA/PEG/OLE films (Figure 3A).

The changes in color can also be noticed through the differentiation in the yellowness index, which not only changed its sign when olive leaf extract was used for PLA films formation, but also substantially increased in comparison with the pristine PLA/PEG film (Figure 3B).

The changes in color of PLA-based films after contact with water, 0.1 M HCl, and 0.1 M NaOH solutions were also evaluated (Table 4). In the case of all of the tested films, contact with all applied media caused darkening (increase in *L* value). Similar observations were made by Michalska-Sionkowska [90] for fish skin collagen material modified with β-glucan. Simultaneously, a reduction of color intensity and yellowness were noticed. Among all CIEL*a*b* color parameters, a more remarkable change was found in the *b* value, which substantially decreased after film swelling. However, no correlation between solution pH and color change could be established.

Two possible phenomena can be responsible for the color changes after swelling. It can be assumed that some of the pigments diffuse from the polymeric matrix into an external solution during swelling. The diffusion strictly correlates with the degree of swelling value and is hindered when low swelling occurs. It should be mentioned that we did not observe extensive swelling in each of the chosen solutions. This observation stays in agreement with the non-ionic nature of PLA and the swelling experiments performed by others [91,92]. Moreover, in contrast with the observations (*L* value), the diffusion of pigments should result in a lightening of PLA/PEG/OLE films. Thus, we assume that the color changes are dominated mainly by the effect of the pH on the stability of the chlorophylls and carotenoids.

### 3.6. Morphology Observations—Scanning Electron Microscopy

**Figure 4:**
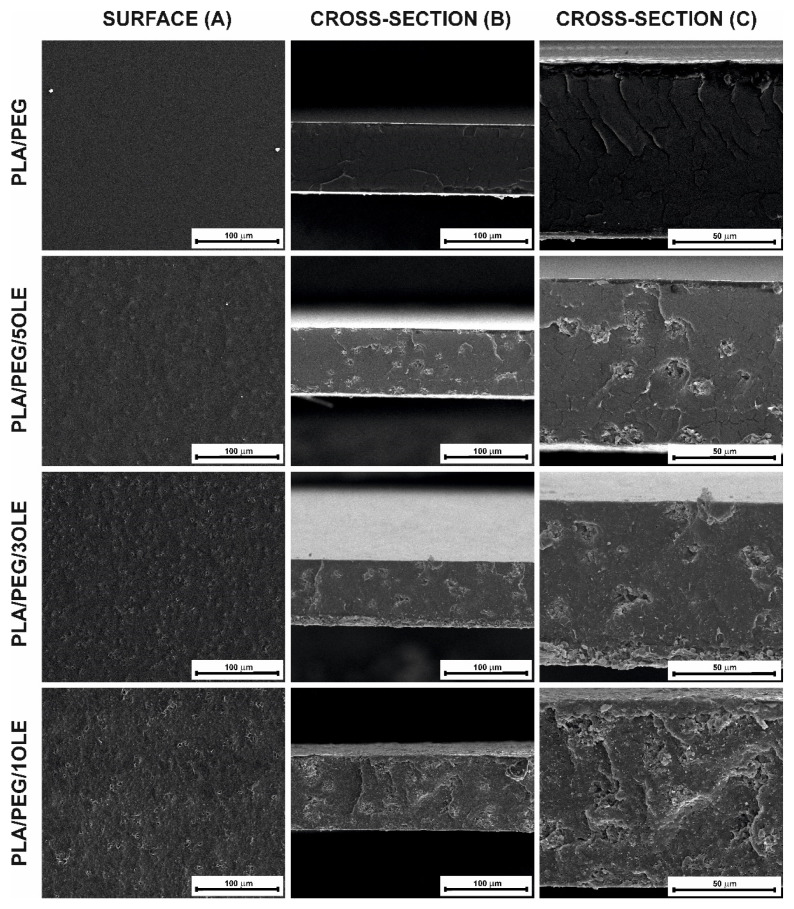
SEM images of PLA/PEG and PLA/PEG films with olive leaf extract addition: surface in 1000× magnification (**A**), and cross-section in 1000× (**B**) and 2500× (**C**) magnification.

The surface and cross-section of the obtained films based on polylactide with olive leaf extract and poly(ethylene glycol) as a plasticizer were observed by Scanning Electron Microscopy. To observe the cross-section, the films were frozen in liquid nitrogen and broken. In Figure 4, surface, and cross-section at 1000× (Figure 4A,B) and cross-section at 2500× (Figure 4C) magnification are presented. It can be seen that surface of polylactide film containing plasticizer (Figure 4A—PLA/PEG) is smooth and flat, without any damage, fissures, or cracks. Similar observation were made in our previous paper [18], for polylactide films with the addition of quercetin and poly(ethylene glycol). Jasim Ahmed et al. [93] also reported a smooth and flat surface of PEG-plasticized polylactide films. These observations suggest good mixing of the polylactide and poly(ethylene glycol) and homogeneity of its blend. Taking into account the cross-section of the PLA/PEG film (Figure 4B—PLA/PEG), cracks can be observed. This may be caused by sample preparation (breaking the film frozen in liquid nitrogen) and the cracking of the gold layer during visualization.

When comparing films without the additive and with the addition of olive leaf extract, significant changes in morphology can be noticed. The surface of PLA/PEG/OLE is folded and inequalities can be observed. With increasing the extract concentration, a more uneven surface of the films was discovered. A similar conclusion was reached by Z. Javidi et al. [77] for polylactide films with the addition of 0.5 and 1.5 % (*w*/*w*) of oregano oil (OOil). It was stated that the addition of a higher amount of OOil into the PLA caused the presence of discontinuities in the resultant films, suggesting that the interaction between oregano oil and PLA was not sufficiently strong to prevent phase separation between both components during the solvent evaporation. The presence of a discontinuous phase in the PLA films with the addition of different essential oils (bergamot, lemongrass, rosemary, and clove) was also observed by Qin et al. [71]. This discontinuous structure would increase the roughness of the films.

Additionally, small holes and cavities were found in the films with OLE addition, as in our previous paper [69] describing the influence of tea tree essential oil on polylactide-based films. However, these surface irregularities were evenly distributed—which might suggest even and reasonably well-mixed ingredients. The incorporation of OLE reduced the compactness of the film. SEM observations stayed in agreement with the FTIR analysis, which did not confirm the strong interactions between the OLE and PLA/PEG film.

When it comes to the thickness of the films—as it can be seen in SEM images (Figure 4B) and in Figure 2D (mechanical properties), where the results for thickness measurements were given—no statistically significant differences in thickness were observed, depending on the concentration of the extract.

## 4. Conclusions

Polymer films with poly(ethylene glycol) as a plasticizer and olive leaf extract were successfully obtained by the solvent evaporation method. It was proven that the addition of OLE positively influences the antioxidant properties of PLA/PEG-based materials. This is due to the composition of the olive leaf extract, which mainly consists of phenolic substances, including oleuropein.

The incorporation of OLE affected the final material crystallinity, which in turn influenced the water vapor permeation rate and mechanical properties. Due to the nature of OLE components, no differences in WVPR between PLA/PEG and PLA/PEG/OLE were observed. The color of the polymeric films was strongly dependent on the presence and content of the extract used and changed during contact with different solutions.

The application of chloroform as a solvent for olive leaf extraction allows for, without additional solvent-evaporation steps, preparing OLE, PLA, and PEG containing film-forming solutions. Thus, the main benefit of the proposed method relies on using the same solvent for both extraction and film formation.

To more strictly characterize the obtained films and to prove their high potential as an active packaging material, additional antimicrobial and storage experiments have already been performed. Their results will be the object of a separate manuscript.

## Figures and Tables

**Table 1:** Phenolic compounds identified by LC-MS/MS using the negative ionization mode of Chemlali olive leaf cultivar extract, including LC-MS/MS parameters and MRM transitions.

N°	TR	M	Exp. ^a^ *m*/*z* for [M-H]^−^	Chemical Formula	λ_max_ [nm]	Main Fragments via MS/MS	Proposed Compound
1	7.5	170.0215	169.0145	C_7_H_6_O_5_	230; 270	125.0243; 124.0168; 97.0294; 73.0190	Gallic acid
2	11.8	154.063	153.0562	C_8_H_10_O_3_	230; 278	123.0453; 109.0291	Hydroxytyrosol
3	18.0	180.0425	179.0351	C_9_H_8_O_4_	295; 324	135.0450; 134.0370; 89.0339	Caffeic acid
4	19.5	640.2003	639.1933	C_29_H_36_O_16_	230; 280; 324	621.1823; 179.0351;1 61.0247	β-hydroxyverbascoside III
5	20.38	610.1534	609.1471	C_27_H_30_O_16_	357	463.0883; 301.0352; 300.0276; 178.9986; 151.0033	Quercetin 3-*O*-rutinoside (rutin)
6	21.1	624.2054	623.1986	C_29_H_36_O_15_	234; 276	461.1652; 315.1071; 179.0349; 161.0242	Verbascoside
7	21.29	448.1006	447.0938	C_21_H_20_O_11_	346	285.0393; 284.0317; 197.0611; 175.0397; 133.0288	Luteolin 7-*O*-glucoside
8	21.8	702.2371	701.2294	C_31_H_42_O_18_	232; 282	539.1762; 437.1430; 377.1238;345.0972; 307.0832; 275.0561; 223.0610; 179.0561; 149.0250; 139.0389; 113.0254	Oleuropein hexoside I
9	22.0	624.2054	623.1986	C_29_H_36_O_15_	234; 278	461.1666; 315.1054; 179.0345; 161.0244	Isoverbascoside
10	23.0	432.1056	431.098	C_21_H_20_O_10_	337	269.0453; 268.0376; 117.0348	Apigenin 7-*O*-glucoside
11	24.2	540.1843	539.1771	C_25_H_32_O_13_	230; 280	403.1236; 377.1241; 371.0979; 327.0880; 307.0822; 275.0920; 223.0610; 179.0703; 165.0560; 149.0245; 139.0401	**Oleuropein I**
12	24.7	540.1843	539.1774	C_25_H_32_O_13_	230; 280	403.1262; 377.1263; 371.1002; 327.0915; 307.0842; 275.0578; 223.0629; 179.0703; 165.0573; 149.0260; 139.0412	**Oleuropein II**
13	25.7	558.2309	557.224	C_26_H_38_O_13_	280	513.2345; 345.1194; 327.1088; 227.1290; 185.1185; 183.0662; 121.0661	6′-*O*-[(2*E*)-2,6-dimethyl-8-hydroxy-2-octenoyloxy]-secologanoside
14	26.3	926.3056	925.2991	C_42_H_54_O_23_	240; 284	893.2633; 763.2456; 745.2360; 693.2030; 539.1765; 521.1655; 377.1239; 307.0823	Jaspolyoside III

TR—time retention [min]; M—molar mass [g/mol]; λ_max_—wavelength of maximum absorbance; *m/z*—the mass-to-charge ratio of the ion.

**Table 2:** The total content of phenolic compounds (TP) and the free radical scavenging activity (*RSA*) of PLA films.

Specimen	TP [mg/mL]	*RSA* [%]
PLA/PEG	-	22.30 ± 2.06
PLA/PEG/1OLE	0.349 ± 0.071	56.26 ± 0.70 *
PLA/PEG/3OLE	0.498 ± 0.106	56.53 ± 1.35 *
PLA/PEG/5OLE	0.637 ± 0.061 ^a^	56.90 ± 3.16 *^#^

^a^—Statistically significant difference vs. PLA/PEG/1OLE; *—Statistically significant difference vs. PLA/PEG (control); ^#^—Statistically significant difference vs. PLA/PEG/1OLE.

**Table 3:** Water Vapor Permeation Rate (WVPR) and the degree of crystallinity ($X_{C}$) of PLA/PEG and OLE containing PLA/PEG films.

Specimen	WVPR [g∙m^−2^∙h^−1^]	$X_{C}$ [%]
Control	42.75 ± 0.48	-
PLA/PEG	10.49 ± 0.81 ^a^	0.72
PLA/PEG/1OLE	10.26 ± 0.65 ^ab^	1.55
PLA/PEG/3OLE	9.07 ± 0.33 ^ab^	1.76
PLA/PEG/5OLE	11.01 ± 1.61 ^ab^	1.61

^a^—statistically significant difference vs. control sample; ^b^—no statistically significant difference vs. PLA/PEG.

**Table 4:** Color (*L*, *a*, *b*, and Δ*E*) parameters of PLA-based films.

Colour Parameter	Conditions	PLA/PEG	PLA/PEG/1OLE	PLA/PEG/3OLE	PLA/PEG/5OLE
	Dry	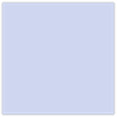	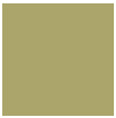	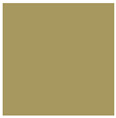	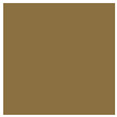
*L*	Dry	86.46 ± 0.05	66.64 ± 1.14	62.78 ± 0.90	50.28 ± 0.60
	HCl *	89.68 ± 0.12	72.60 ± 0.27	75.24 ± 1.14	58.70 ± 0.39
	Water *	89.42 ± 0.16	79.56 ± 0.08	67.52 ± 0.50	61.48 ± 0.84
	NaOH *	89.32 ± 0.07	71.34 ± 0.45	72.26 ± 0.79	70.02 ± 0.60
*a*	Dry	0.76 ± 0.05	−4.06 ± 0.08	−0.30 ± 0.13	5.34 ± 0.10
	HCl *	0.74 ± 0.10	−4.26 ± 0.08	−4.04 ± 0.08	−0.70 ± 0.17
	Water *	0.82 ± 0.04	−3.34 ± 0.05	−2.80 ± 0.14	−2.72 ± 0.19
	NaOH *	1.12 ± 0.04	−2.62 ± 0.04	−3.12 ± 0.15	−1.02 ± 0.04
*b*	Dry	−12.68 ± 0.10	29.08 ± 0.85	30.42 ± 0.16	28.64 ± 0.63
	HCl *	−13.36 ± 0.05	17.12 ± 0.29	15.34 ± 1.03	19.68 ± 0.18
	Water *	−13.28 ± 0.07	10.98 ± 0.07	20.84 ± 0.05	20.80 ± 0.15
	NaOH *	−13.88 ± 0.07	19.64 ± 0.54	22.78 ± 0.22	20.00 ± 1.07
ΔE	Dry	-	46.50 ± 0.32	49.20 ± 0.30	55.12 ± 0.23
	HCl *	-	35.30 ± 0.19	32.51 ± 0.52	45.32 ± 0.20
	Water *	-	26.52 ± 0.04	40.71 ± 0.25	44.22 ± 0.43
	NaOH *	-	38.23 ± 0.26	40.66 ± 0.19	39.06 ± 0.63

*—after immersing in HCl/Water/NaOH media.

## Data Availability

The data presented in this study are available on request from the corresponding author.

## Supplementary Materials

The following are available online at https://www.mdpi.com/article/10.3390/ma14247623/s1, Figure S1. Base peak chromatogram of Chemlali olive cultivar leaf extract, Figure S2. DSC thermograms of PLA/PEG and PLA/PEG/OLE films, Figure S3. Real photo of fresh olive leaf extracts in chloroform, Table S1. DSC thermal data of PLA/PEG and PLA/PEG/OLE films.
