# Supplementary material for: Polylactide Films with the Addition of Olive Leaf Extract—Physico-Chemical Characterization
Source: Materials (Basel). 2021 Dec 11;14(24):7623. doi: 10.3390/ma14247623 (PMC8706180; doi:10.3390/ma14247623)
Supplement: Supplementary file 1 [file materials-14-07623-s001.zip › materials-1496536-supplementary.pdf]

materials

Supplementary Materials

Polylactide Films with the Addition of Olive Leaf Extract—Physico-Chemical Characterization

Sylwia Grabska-Zielińska [1,*], Magdalena Gierszewska [1,*], Ewa Olewnik-Kruszkowska [1] and Mohamed Bouaziz [2]

1 Department of Physical Chemistry and Physicochemistry of Polymers, Faculty of Chemistry, Nicolaus Copernicus University in Toruń, Gagarin 7 Street, 87-100 Toruń, Poland; olewnik@umk.pl
2 Electrochemistry and Environmental Laboratory, National Engineering School of Sfax, University of Sfax, BP1173, Sfax 3038, Tunisia; mohamed.bouaziz@fsg.rnu.tn
* Correspondence: sylwia.gz@umk.pl (S.G.-Z.); mgd@umk.pl (M.G.)

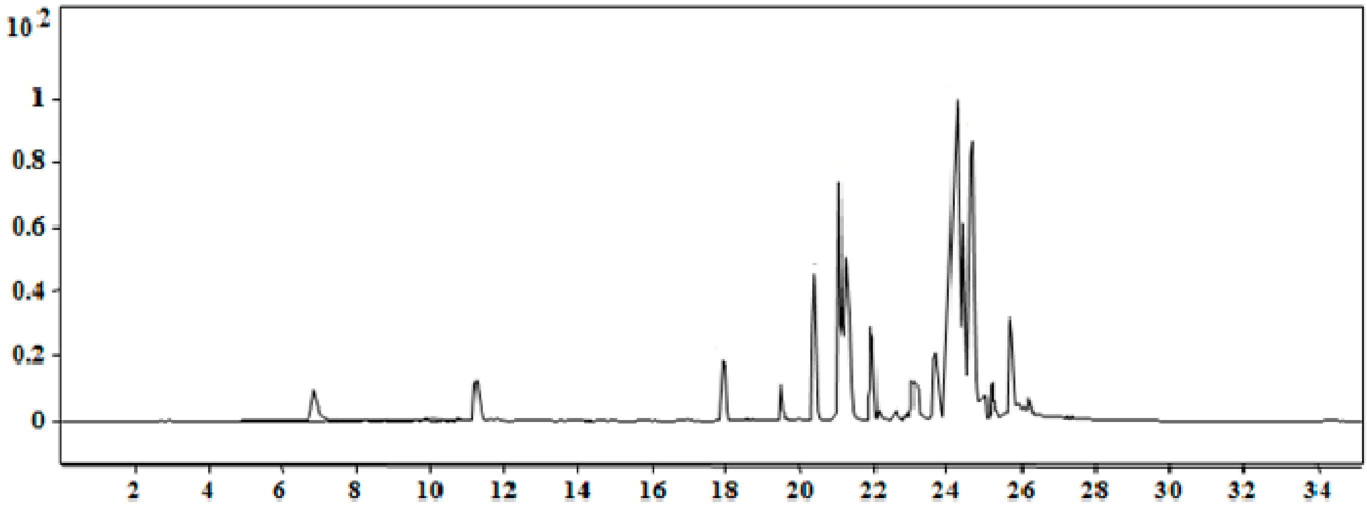

Figure S1. Base peak chromatogram of Chemlali olive cultivar leaf extract.

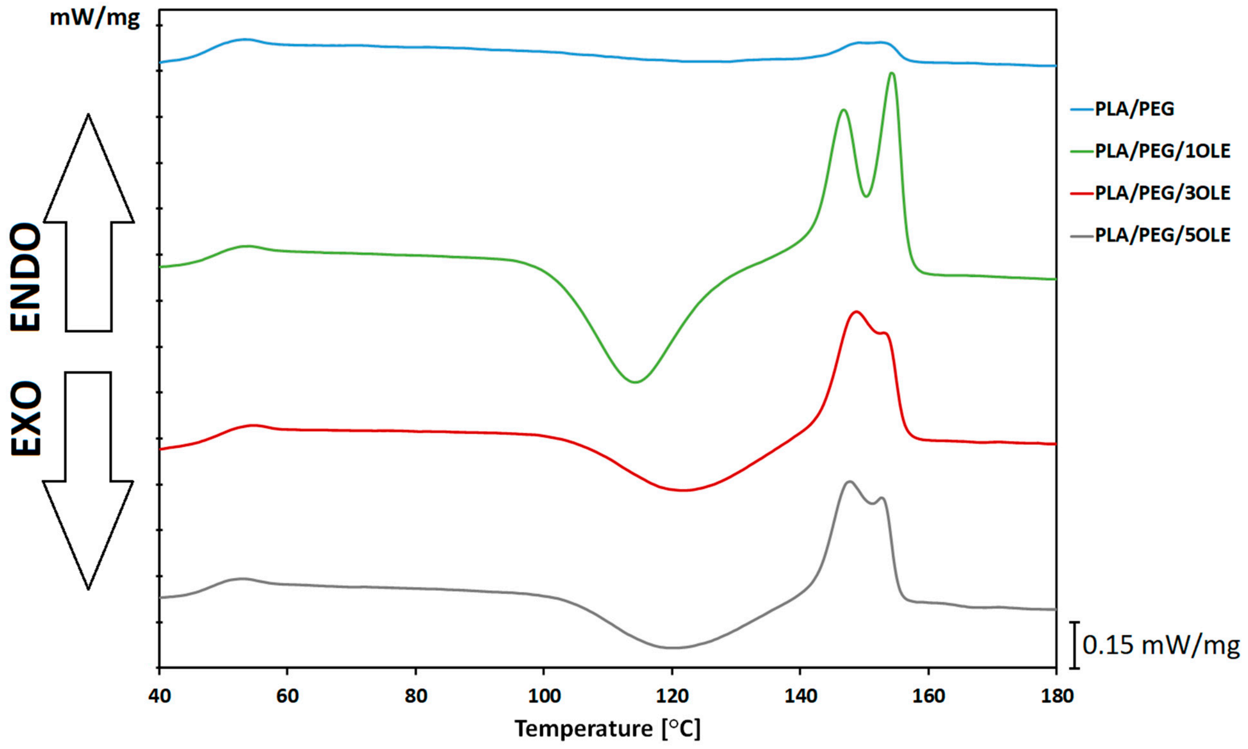

Figure S2. DSC thermograms of PLA/PEG and PLA/PEG/OLE films.

Table S1. DSC thermal data of PLA/PEG and PLA/PEG/OLE films.

**Specimen**	ΔH_m **[J·g⁻¹]**	ΔH_{cc} **[J·g⁻¹]**	X_C **[%]**
PLA/PEG	2.50	3.26	-
PLA/PEG/1OLE	36.83	38.39	1.55
PLA/PEG/3OLE	22.39	24.06	1.76
PLA/PEG/5OLE	20.77	22.21	1.61

ΔH_m – melting enthalpy, ΔH_{cc} - enthalpy of cold crystallization

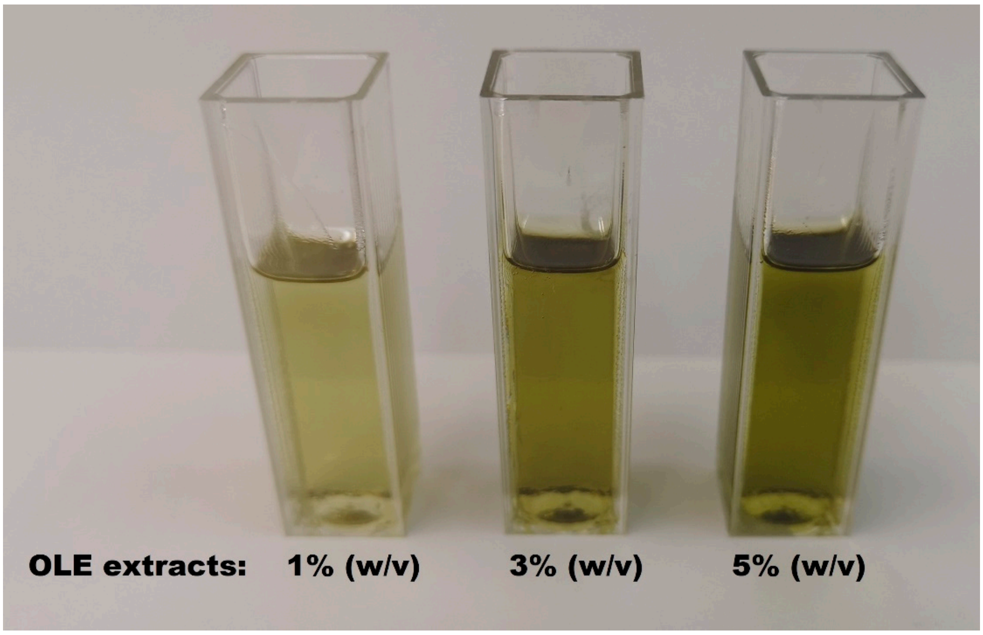

Figure S3. Real photo of fresh olive leaf extracts in chloroform.
